# New lucinid bivalves from shallow and deeper water of the Indian and West Pacific Oceans (Mollusca, Bivalvia, Lucinidae)

**DOI:** 10.3897/zookeys.326.5786

**Published:** 2013-08-26

**Authors:** John D. Taylor, Emily A. Glover

**Affiliations:** 1 Department of Life Sciences, The Natural History Museum, London SW7 5BD, UK

**Keywords:** Bivalvia, Lucinidae, chemosymbiosis, taxonomy, new species, deep water, Indo-West Pacific

## Abstract

Four new species and a new genus of lucinid bivalves are described from shallow and deeper waters in the Indian and West Pacific Oceans. The new genus *Scabrilucina* (subfamily Lucininae) includes the little-known *Scabrilucina victorialis* (Melvill, 1899) from the Arabian Sea and *Scabrilucina vitrea* (Deshayes, 1844) from the Andaman Sea as well as a new species *Scabrilucina melvilli* from the Torres Strait off northeastern Australia. *Ferrocina brunei* new species (Lucininae) was recovered from 60 m near oil drilling activities off Borneo; its anatomy confirmed the presence of symbiotic bacteria. Two unusual deeper water species of Leucosphaerinae are described, both species included in on-going molecular analyses; *Gonimyrtea ferruginea* from 400–650 m in the southwest Pacific and *Myrtina reflexa* from 200–825 m off Zanzibar and Madagascar.

## Introduction

Over the last 20 years there has been much taxonomic interest in the chemosymbiotic bivalve family Lucinidae with many new genera and species described from shallow to bathyal depths of the tropical Indo-West Pacific (Taylor and Glover 1997, 2002, 2005; Glover and Taylor 2001, 2007, 2008; Glover et al. 2008; Oliver and Holmes 2006; Bouchet and Cosel 2004, Cosel and Bouchet 2008, Okutani 2011, Glover and Taylor in press). Despite this activity there remain many undescribed or unregarded species whose systematic position is obscure.

In this paper a new genus is introduced to accommodate *Lucina victorialis* (Melvill, 1899) previously known from just a few valves collected in the Arabian Sea in the late 1800s but classified initially as *Cryptodon* (i.e. Thyasiridae) because of the deep sulcus and trigonal shape. Discovery in the Museum of Comparative Zoology, Harvard University of a large sample collected in the Arabian Sea as part of the 1963 International Indian Ocean Expedition has allowed re-description and assessment of the unusual features of this species. We also include within this genus the neglected species *Lucina vitrea* Deshayes, 1844, with new records from Southeast Asia, and another species with similar characters is newly described from the Torres Strait off northeastern Australia.

Benthic sampling at about 60 m depth off Brunei in north Borneo recovered from near oil drilling activities an unusual, small, mottled red-brown lucinid that on shell characters can be classified as a new species of *Ferrocina* a genus previously known from the rare type species *Ferrocina multiradiata* Glover & Taylor, 2007 from off Fiji and New Caledonia and another species from the Philippines (Glover and Taylor in press). Formalin preserved animals were available and we include some anatomical information.

In a molecular analysis of Lucinidae we included an individual of an undescribed genus and species dredged from over 600 m on the Chesterfield Bank west of New Caledonia (UGS-3 in Taylor et al. 2011, fig. 7J). This taxon grouped with a cluster of other genera in the subfamily Leucosphaerinae and we now classify and describe it as a new species of *Gonimyrtea* Marwick, 1929. Also in the Leucosphaerinae we describe a new species of *Myrtina* Glover & Taylor, 2007 from off Zanzibar and Madagascar between 200–800m and for which molecular data is available.

## Institutional abbreviations

AM Australian Museum, Sydney

MCZ Museum of Comparative Zoology, Harvard University

NHMUK The Natural History Museum, London

MNHN Muséum national d’Histoire Naturelle, Paris

UMC Zoological Museum, University of Copenhagen

USNM United States National Museum of Natural History

### Other abbreviations

H shell height

IWP Indo-West Pacific

L shell length

LV left valve

PI protoconch I length

PII protoconch II length

pv paired valves

RV right valve

T tumidity of single valve

v valve (s)

## Systematics

### Family Lucinidae Fleming, 1828
Subfamily Lucininae Fleming, 1828

#### Scabrilucina gen. n.

http://zoobank.org/78644155-2436-4683-8C37-DB6E3A00A0AC

http://species-id.net/wiki/Scabrilucina

##### Type species.

*Cryptodon victorialis* Melvill, 1899. Here designated. Northern Indian Ocean.

##### Diagnosis.

Small to medium size, L to 40 mm, thin, semi-translucent, usually slightly higher than long, ovoid to subtrigonal, strong posterior sulcus with broad sinus at posterior margin. Sculpture of sharp, fine, commarginal lamellae. Ligament short, set in shallow resilifer. Hinge with small to vestigial cardinal teeth in both valves, lateral teeth usually absent but small anterior lateral tooth may be present in juvenile shells. Anterior adductor muscle scar long, thin, ventrally detached from pallial line for ½–¾ of length, dorsal part runs on to hinge. Interior shell with translucent spots.

##### Etymology.

Derivedfrom Latin *scaber* rough and *Lucina*, reference to the rough surface formed by closely spaced, sharp, commarginal lamellae. Feminine.

##### Comparison with other genera.

*Scabrilucina* can be readily distinguished from other lucinids by the subtrigonal outline with a prominent posterior sulcus, the fine, regular, sharp-edged, commarginal lamellae and the absence of radial sculpture. No preserved samples of *Scabrilucina* were available for molecular analysis but a small lucinid from the Philippines with some similar characters was included in a prior analysis (Taylor et al. 2011) as ‘*Lucina*’ *desiderata* Smith, 1885. We now consider this a new species in a separate genus (Glover and Taylor in press) and related to *Scabrilucina*. Evidence from 18 S and 28 S rRNA genes places ‘*Lucina*’ *desiderata* in the subfamily Lucininae that by inference also includes *Scabrilucina*.

The little known genus *Semelilucina* Cosel & Bouchet, 2008, type species *Semelilucina semeliformis* from offshore muddy habitats in Tanimbar, SE Indonesia, may be related to *Scabrilucina*. The two genera differ in shell shape but have similar sculpture of fine, regularly spaced, sharp commarginal lamellae. Cosel and Bouchet 2008 considered *Semelilucina* closely related to *Dulcina* (Leucosphaerinae) and we followed this in our classification (Taylor et al. 2011). However, shell morphology suggests that *Semelilucina* is better placed in the Lucininae.

#### Scabrilucina victorialis

(Melvill, 1899)

http://species-id.net/wiki/Scabrilucina_victorialis

Figs 1 –2 , 3A

Cryptodon victorialis Melvill, 1899: 98-9, pl. 2, figs 9, 9a.
Loripes victorialis – Smith 1906: 256.
Loripes victorialis – Melvill and Standen 1907: 815.
Lucina victorialis – Oliver 1995: 235, fig. 1024.

##### Type material.

Holotype, left valve (NHMUK 1899.12.18.28), H 25.3 mm, L 23.5 mm.

##### Type locality.

Melvill (1899) p. 99 states ‘near Karachi, and also Malcolm Inlet nr Muscat, Oman 24 fathoms’ (44 m) (Malcolm Inlet is Ghubbat al Ghazira).

##### Other material examined.

**Northern Arabian Sea:** 141 valves (MCZ 362493), 47 miles E of Duhat Sharjah, Oman, Arabian Sea, 50.5–52 fthms (92–95 m), Anton Brunn Cruise, 4b stn 255A (25°50'N, 57°07'E, 30 November 1963). 2 valves (USNM 716871), localityas above, Anton Brunn station 255A. 1 valve (NHMUK 99.2.18.10)25 fthms (45.7 m), 26°23'N, 54°53'E, Melvill collection. 1 valve (NHMUK) off Gwadhur (Gwadar), Pakistan, 70 fthms (128 m), Townsend collection. 4 valves, NHMUK, Gulf of Oman, Townsend collection. 1 valve (NHMUK 1906.10.12.90) Arabian Gulf, 47 fthms, Investigator station 346, (Smith 1906).

##### Description.

**Figure 1.**
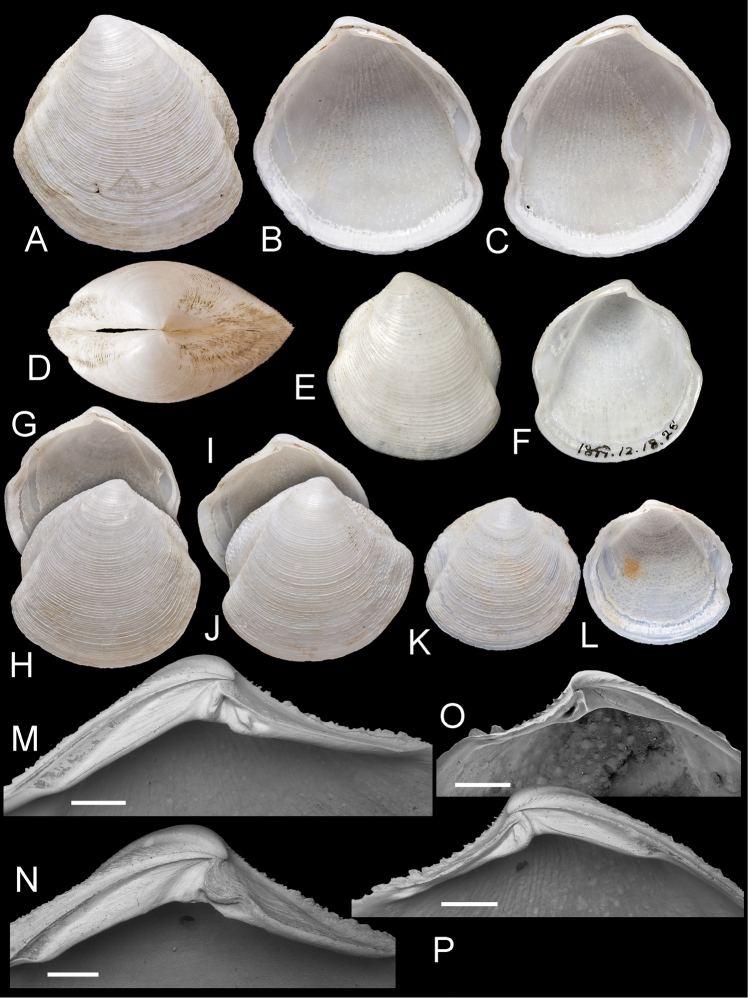
*Scabrilucina victorialis* (Melvill, 1899) except where otherwise stated all specimens MCZ 362493. **A–C** exterior of left valve and interiors of right and left valves, L = 40.4 mm **D** Dorsal view of **A–C E–F** Holotype *Cryptodon victorialis* Melvill, 1899, NHMUK 1899.12.18.28 exterior and interior of left valve, L = 23.5 mm **G–H** Interior and exterior of right valve L = 32.1 mm **I–J** Interior and exterior of right valve, L = 36.2 mm **K–L** Exterior and interior of juvenile right valve, L= 22.9 mm **M** Detail of hinge teeth of left valve. Scale bar = 1 mm **N** Hinge of left valve Scale bar = 1 mm. **O** Hinge of right valve juvenile shell, NHMUK Scale bar 1 mm **P** Hinge of right valve. Scale bar = 1 mm.

**Figure 2.**
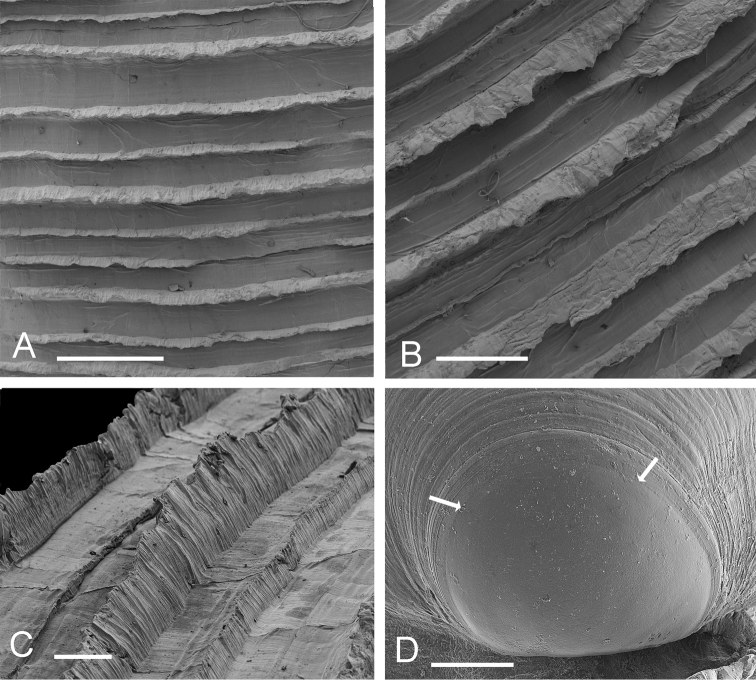
*Scabrilucina victorialis* shell features. **A–C** MCZ 362493 **A** Commarginal lamellae. Scale bar = 1 mm. **B** Detail of commarginal lamellae. Scale bar = 500 µm. **C** Detail of commarginal lamellae. Scale bar = 200 µm. **D** Protoconch NHMUK. Arrows mark boundary between PI and PII. Scale bar = 50 µm.

Shell white, thin, often semi-translucent, H to 43 mm, L to 39 mm, higher than long, H/L = 1.03±SD 0.03 (n=17), moderately inflated T/L 0.27±SD 0.014 (n=17), outline subtrigonal in larger shells, juvenile shells subcircular. Posterior sulcus, prominent, deeply incised, with marginal sinus; anterior sulcus narrow and shallow. Sculpture of fine, closely spaced (200–500 µm apart), thin, sharp-edged, striated, commarginal lamellae (Figs 2A–C) that narrow to around 30 µm at the distal edges and are slightly elevated along the posterior and anterior dorsal margins. Fine sediment is frequently trapped between lamellae. Protoconch (Fig. 2D): PI 150 mm, PI + PII 165 µm, PII with 2-3 growth increments. Lunule long, lanceolate and asymmetric, slightly larger in left valve. Ligament short, set in narrow groove. Hinge plate narrow, LV with 2 cardinal teeth, the anteriormost reduced and often obscured by ventral extension of lunule (Fig. 1M–P), lateral teeth absent; RV with 1-2 small cardinal teeth, sometimes obscure, small anterior lateral tooth sometimes visible in younger shells (Fig. 1P). Anterior adductor muscle scar long, thin, tapering at ventral tip, detached for ½–⅔ length and diverging ventrally at angle of 25–30° from pallial line, posterior adductor scar ovate. Pallial line broad, continuous, inner shell surface rough with many small translucent circular spots representing mantle attachment points and prominent radial grooves. Track of pallial blood vessel visible. Inner shell margin smooth.

**Figure 3.**
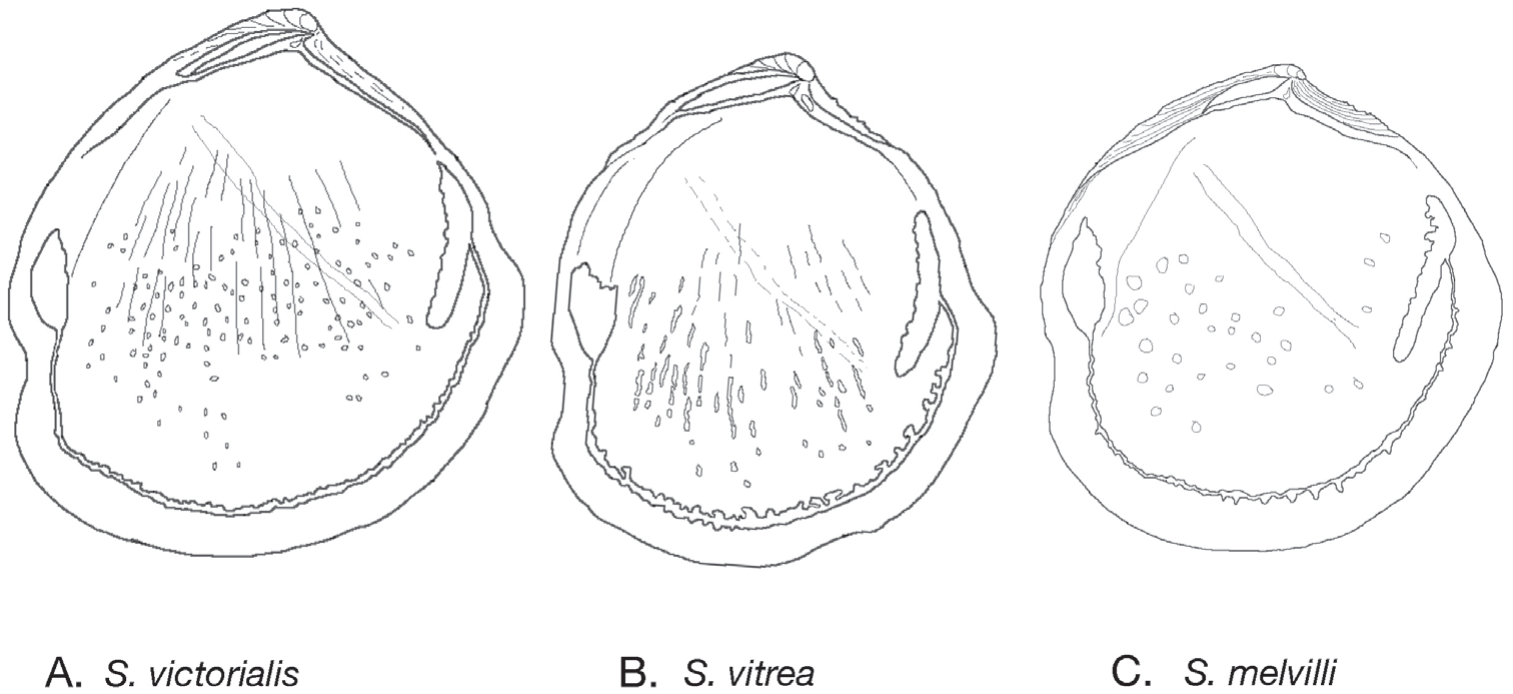
Internal drawings *Scabrilucina* species. **A** S. *victorialis* **B** *Scabrilucina vitrea* **C** *Scabrilucina melvilli*.

##### Distribution.

**Figure 4.**
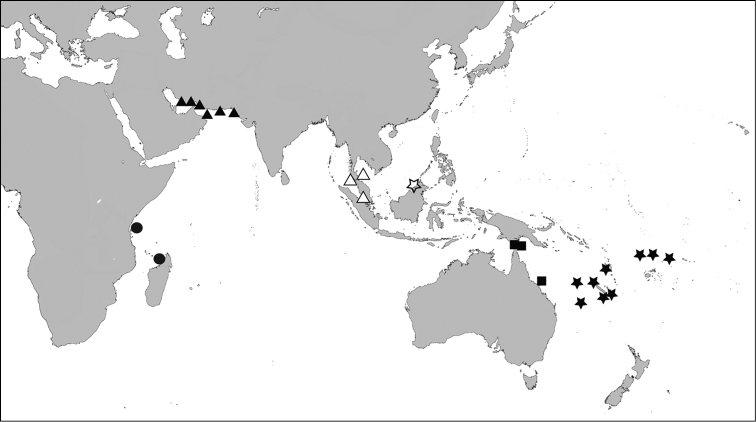
Distribution map - *Scabrilucina victorialis*, solid triangles; *Scabrilucina vitrea*, open triangles; *Scabrilucina melvilli*, solid squares; *Gonimyrtea ferruginea*, solid stars; *Myrtina reflexa*, solid circles, *Ferrocina brunei* open star.

50 – ca 150 m in offshore muds, northern Arabian Sea, Gulf of Oman, northeastern Arabian Gulf (Fig. 4). Smith (1906) records *victorialis* from the Arabian Gulf, 47 fthms, Investigator station 346, 26°37'30"N, 53°03'30"E.

##### Remarks.

**Figure 5.**
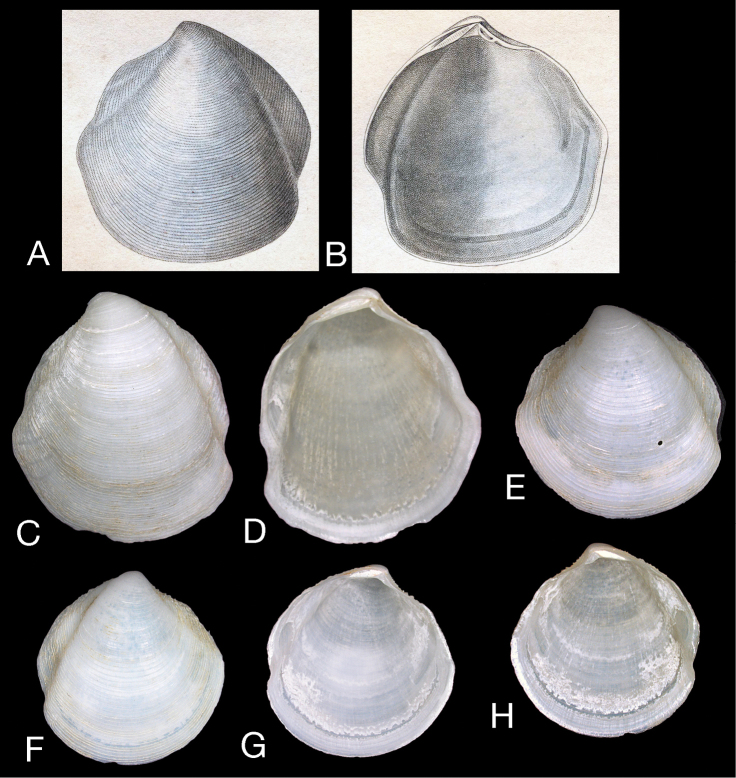
*Scabrilucina vitrea* (Deshayes, 1844) **A–B** Scanned images of the original illustrations of *Lucina vitrea* from Deshayes, 1844 pl. 106, length 22 mm **C–H** specimens from Andaman Sea, Thailand (ZMC) **C–D** Exterior and interior left valve L = 21.9 mm **E** Exterior of left valve, L = 16.8 mm **F–H** Exterior of RV, interiors of left and right valves, L = 16.8 mm.

This species is known only from shells. *Scabrilucina victorialis* is characterised by the deep cleft of the posterior sulcus and the fine, sharp, commarginal lamellae. *Scabrilucina vitrea* (see below) from off Sumatra and Gulf of Thailand is smaller, taller, and thinner shelled. The shell shape and the deep posterior sulcus superficially resemble some Thyasiridae such as *Conchocele* and this influenced Melvill’s initial placement in *Cryptodon*. *Scabrilucina victorialis* is also similar to *Scabrilucina melvilli* (new species below) from Australia that is distinguished by its smaller size, less deeply incised posterior sulcus and more widely spaced commarginal lamellae. Many of the shells of *Scabrilucina victorialis* from off Oman (MCZ 362493) and *Scabrilucina vitrea* from Thailand are penetrated by narrow, straight-sided holes ca 450 µm diameter (Figs 1A, C, 5E) comparable with those resulting from octopus predation (Cortez et al. 1998, Todd and Harper 2010).

#### Scabrilucina vitrea

(Deshayes, 1844)

http://species-id.net/wiki/Scabrilucina_vitrea

Figs 3B , 5

Lucina vitrea Deshayes 1844: pl. 106.

##### Type material.

Not located.Original descriptionL 22 mm, H 20 mm.

##### Type locality.

‘les mers de Sumatra’

##### Material examined.

**Thailand:** 2 paired valves and 5 valves (ZMC),Andaman Sea, 27-35 m, 7°00'15"N, 99°21'42"E, 5^th^ Thai Danish Expedition stn 1052 & 1054, sandy mud with dead shells, 10 February 1966. 1 valve (ZMC) Gulf of Thailand, 30 fthms (54 m) 10°04.10'N, 100°10.1E, Naga Expedition 1960, stn 60–853. **Malaysia:** 1 valve (NHMUK 163529), Malacca, Cuming Collection.

##### Description.

Shell white, L to 22.0 mm, H to 25.1 mm, slightly higher than long, H/L 1.08 ± SD 0.05 (n=5), very thin shelled, translucent, prominent posterior and anterior sulcus, umbones prominent. Sculpture of fine, thin commarginal lamellae, lamellae slightly irregular and elevated at posterior and anterior dorsal area, radial sculpture absent. Lunule broad, lanceolate, slightly impressed. Hinge extremely narrow, right valve with single cardinal tooth, lateral teeth absent, left valve with 2 small cardinal teeth, lateral teeth absent. Anterior adductor scar long, narrow, pointed, detached for 2/3rds of length, posterior scar ovoid, pallial line entire with dorsal elevations, close to shell margin, inner shell surface with many fine points of mantle attachment, shell surface glossy outside the pallial line.

##### Distribution.

Andaman Sea, Straits of Malacca and Gulf of Thailand (Fig. 4).

##### Remarks.

Similar to *Scabrilucina victorialis* but smaller and less trigonal in outline of the adult shell, with finer commarginal sculpture and thinner shell.

The type material of *Scabrilucina vitrea* has not been located but the original figures of Deshayes are clear (Figs 5A, B). The type locality of Sumatra is close to that of the other samples mentioned here. As far as we are aware, except for a listing in Tryon (1872), this species name has not been mentioned since the original description in 1844.

#### Scabrilucina melvilli sp. n.

http://zoobank.org/EC1E8F2B-0F9E-4DE1-8DE7-4C26695A61FB

http://species-id.net/wiki/Scabrilucina_melvilli

Figs 3C , 6

##### Type material.

*Holotype*: 1 whole shell (AM C. 360708), H 21.2 mm, L 21.0, T (single valve) 5.2 mm. *Paratypes*: single left valve, H 20.5mm, L 18.3 mm, juvenile shells 4 LV and 4 RV, locality as holotype (AM C. 479181).

##### Type locality.

Northeastern Australia,Queensland, E of Bowen 19°45.7'S, 148°19'E, 46 m, thin, grey mud, coll. PH Colman & F Rowe.

##### Other material examined.

Australia, Queensland: 6 valves (AM C. 036166), Albany Passage, Cape York Peninsula, 10°45'S, 142°37'E, 4–14 fthms (7–26 m), mud & sand, coll. C. Hedley & A. Mc Culloch. (3 LV, 3 RV+ juveniles. Left valves – H 26.3 mm, L 24.3 mm; H 24.3 mm, L 23.2 mm; H 23.3 mm, L 20.9 mm; right valves - 19.0 mm L 18.6 mm; H 18.7 mm, L 17.2 mm). 1 valve (AM C. 360707), Horn Island, Torres Strait, 10°35.6S, 142°14.6'E, mangroves and sand flats, near jetty, coll. W.F. Ponder & I. Loch. (1 LV H 18.6 mm, L 17.3 mm).

##### Description.

**Figure 6.**
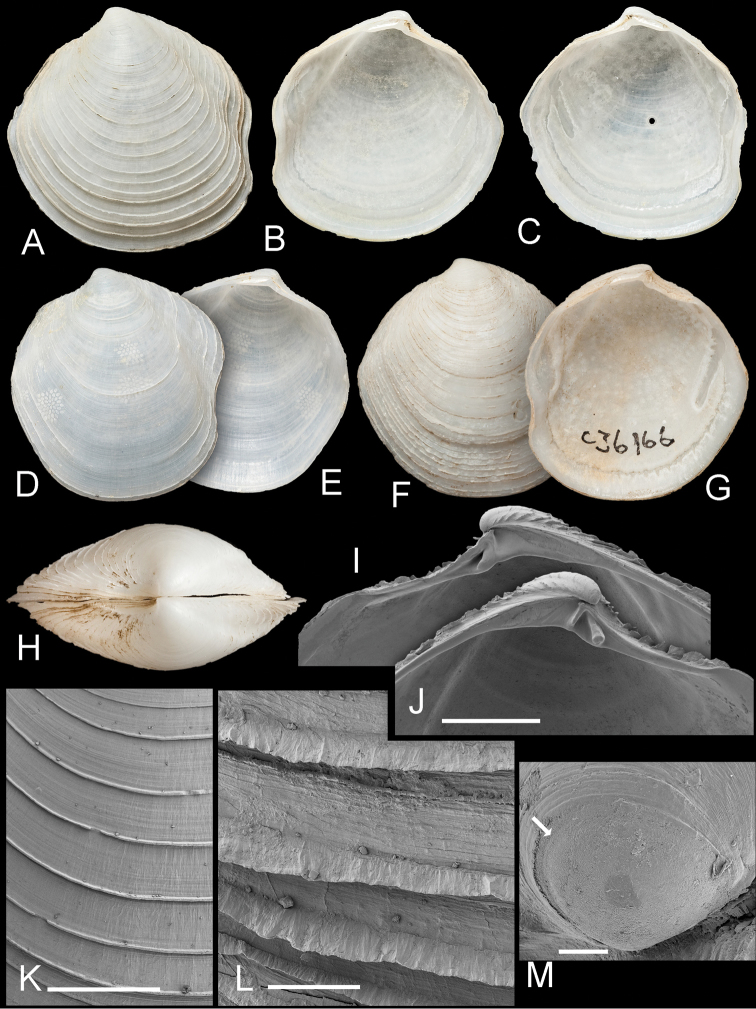
*Scabrilucina melvilli* sp. n. **A–C** Holotype AMS C. 360708 exterior of left valve and interior of left and right valves, L = 21 mm **D–E** paratype AMS C. 360708 exterior and interior of left valve, L= 18.3 mm **F–G** paratype AMS 036166 exterior and interior of left valve, L= 23.1 mm **H** Dorsal view of holotype **I–J** Detail of hinge teeth of right and left valves of juvenile shells. Scale bar = 1 mm **K** External sculpture. Scale bar = 1 mm **L** Detail of commarginal lamellae. Scale bar = 200 µm **M** Protoconch. Arrow marks boundary between PI and PII. Scale bar = 50 µm.

Shell fragile, thin, white, semi-translucent, subtrigonal, L to 26.3 mm, H to 26.3 mm, higher than long H/L = 1.05 ± SD 0.04 (n = 9), moderately inflated T/L = 0.27 ± 0.02 (n = 9). Posterior sulcus broad, shallow with marginal sinus. Anterior dorsal area weakly defined. Sculpture (Fig. 6K, L) of regularly spaced, low, thin, sharp, commarginal lamellae with faint radial folds in interspaces. Commarginals more closely spaced in larger specimens. Commarginal lamellae elevated to scales along dorsal margin. Protoconch (Fig. 6M): 150 µm, PII a narrow rim. Lunule short, lanceolate, with more prominent growth increments in LV. Ligament short, set in groove. Hinge plate narrow (Fig. 6I, J), LV with two small cardinal teeth, anterior tooth larger, RV with single cardinal tooth, in larger specimens teeth sometimes obscure. Small anterior lateral present in RV of juveniles. Anterior adductor scar long, thin, detached ventrally from pallial line for ¾ of length at angle of 25°. Posterior adductor scar ovoid. Pallial line narrow, entire. Shell inside pallial line with small round points of mantle attachment, radial grooves, track of pallial blood vessel visible as shallow groove. Shell margin smooth.

##### Distribution.

Torres Strait, northeastern Australia (Fig. 4) shallow water mud to 50 m.

##### Remarks.

See above for comparison with *Scabrilucina victorialis*.

##### Etymology.

Named for James Cosmo Melvill (1845–1929), British malacologist who described many IWP species. Noun in genitive case.

#### Ferrocina

Glover & Taylor, 2007

http://species-id.net/wiki/Ferrocina

##### Type species.

*Ferrocina multiradiata* Glover & Taylor, 2007 by original designation.

##### Diagnosis.

Shell to 20 mm, thin, subovate, posteriorly truncate, sculpture of numerous fine, often indistinct radial ribs crossed by fine commarginal threads. Hinge plate thin, small single cardinal tooth in RV, two cardinals in LV, lateral teeth very small or obsolete. Anterior adductor scar short, detached for 1/3 of length. Interior shell margin finely to coarsely dentate. Colour pale orange to rusty red-brown, sometimes blotchy.

##### Remarks.

The unusual and rare genus *Ferrocina* was first recognised from Vanuatu, Fiji and New Caledonia at depths from 80–400 m (Glover and Taylor 2007) and is known from a few shells of the type species. A second species has been identified from the Philippines (Glover and Taylor in press) also from a few shells. From shell features including the dentition and presence of a pallial blood vessel scar we classify this genus in the Lucininae. Additionally the ramshorn shaped visceral extension is also seen in the lucinine *Bathyaustriella* (Glover et al. 2004).

#### Ferrocina brunei sp. n.

http://zoobank.org/1954D59B-0F70-481B-9FE9-159FDF23DD24

http://species-id.net/wiki/Ferrocina_brunei

Figs 7 , 8 , 9C

##### Type material.

*Holotype*: whole shell NHMUK 20130122 L 8.2 mm, H 6.7 mm, T 1.6 mm; *Paratypes*: NHMUK 20130123, figured L 8.4 mm, H. 7.3 mm, T 2.1 mm; L 8.9 mm, H 7.8 mm, T 2.1 mm, non figured 19 v.

##### Type locality.

**Brunei,** 05°21'12"N, 111°26'21"E, 63 m, muddy sand near oil drilling rig.

##### Description.

**Figure 7.**
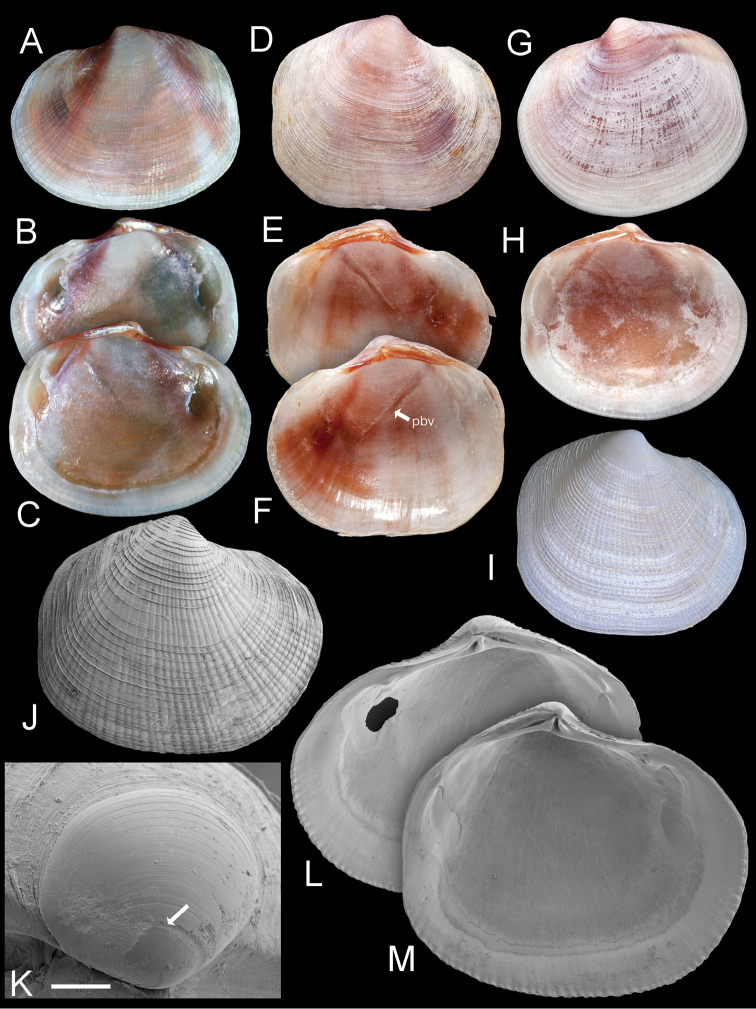
*Ferrocina brunei* sp. n. **A–C** Holotype NHMUK 20130122 Exterior of left valve and interior of right and left valves, L = 8.2 mm **D–F** Paratype NHMUK 20130123 exterior of right valve and interior of left and right valves, L = 8.4 mm. pbv trace of pallial blood vessel **G–H** Paratype NHMUK 20130123 exterior and interior of left valve, L = 8.9 mm **I** Exterior of right valve of white form NHMUK 20130123, L = 7.9 mm **J** SEM of right valve L = 6.0 mm **K** Protoconch, arrow at PI /PII junction. Scale bar = 50 µm **L–M** Interior of right and left valves L = 5.2 mm.

Shell small, H to 7.8 mm, L to 8.9 mm, T to 2.1 mm, longer than high H/L = 0.89 ± 0.034 (n=13). Colour grey-white with patches, streaks or stripes of rusty red, more pronounced dorsally, including lunule, occasionally whole shell red-brown; internally red brown particularly at anterior. Sculpture of numerous (ca 40) low, radial ribs that divide and intercalate, crossed by very fine, widely spaced commarginal lamellae. Shallow posterior sulcus and posterior dorsal area with slightly elevated commarginal lamellae, radial ribs absent. Anterior dorsal area also without ribs. Protoconch (Fig. 7K) PI = 82 µm, PI+ PII = 159 µm, PII with many growth increments. Lunule short lanceolate, slightly impressed, asymmetrical, greater part in right valve, brown coloured. Ligament set in shallow groove. Hinge plate thin (Figs 7L, M), RV with single cardinal tooth and small anterior and posterior lateral teeth; LV with two cardinal teeth, anteriormost is larger and faint sockets for anterior and posterior lateral teeth. Anterior adductor muscle scar broad, short, detached for ½ of length at an angle of 25°. Pallial blood vessel trace prominent, terminates ventral to anterior adductor scar. Pallial line entire. Inner shell margin denticulate.

##### Anatomy.

**Figure 8.**
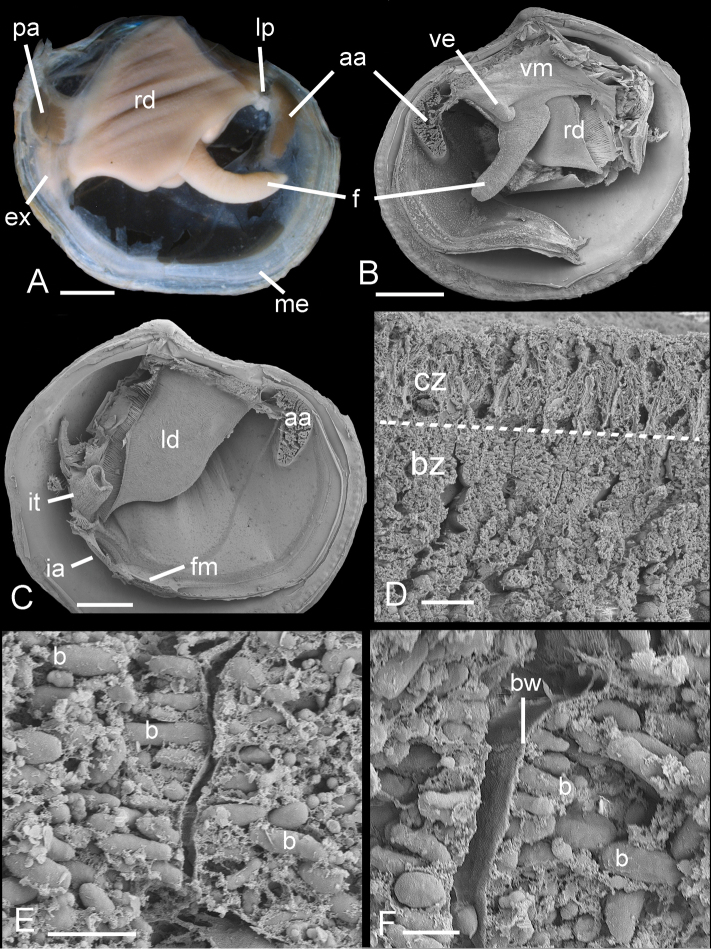
*Ferrocina brunei* sp. n. **A** Body from right side. Scale bar = 1 mm **B** Body from left side, mantle and left demibranch removed showing foot and visceral extension. Scale bar = 1 mm **C** Body from right side with visceral mass, right demibranch and mantle removed. Scale bar = 1 mm **D** Section through part of demibranch showing ciliated and bacteriocyte zones. Scale bar = 20 µm **E** Part of ctenidial filament in bacteriocyte zone showing bacteria. Scale bar = 5 µm **F** Detail of bacteriocytes and bacteria aligned normal to the apical cell wall. Scale bar = 2 µm. aa, anterior adductor muscle. b, bacteria. bw, bacteriocyte apical wall. bz, bacteriocyte zone. cz, ciliated zone. ex, exhalant aperture. f, foot. fm, fused mantle. ia, inhalant aperture. it, inverted tube of posterior exhalant aperture. ld, left demibranch. lp, labial palps. me, mantle edge. pa, posterior adductor muscle. rd, right demibranch. ve, visceral extension. vm, visceral mass.

Ctenidia comprising inner demibranchs (Fig. 8A), pink, thick, occupying about ½ of mantle cavity. Foot cylindrical with small heel. Labial palps small ridges. Visceral mass anterior to foot laterally extended into pair of ramshorn-like coiled structures (Fig. 8B). Posterior exhalant aperture with inverted tube (Fig. 8C), inhalant aperture with small papillae, short section of fused mantle ventral to aperture. Ctenidial filaments with thick bacteriocyte zone with bacteriocytes packed with short rod-shaped bacteria 2-5 µm long and 1-2 µm wide (Figs 8D–F). Bacteria aligned with long axes normal to apical surfaces of bacteriocytes.

**Figure 9.**
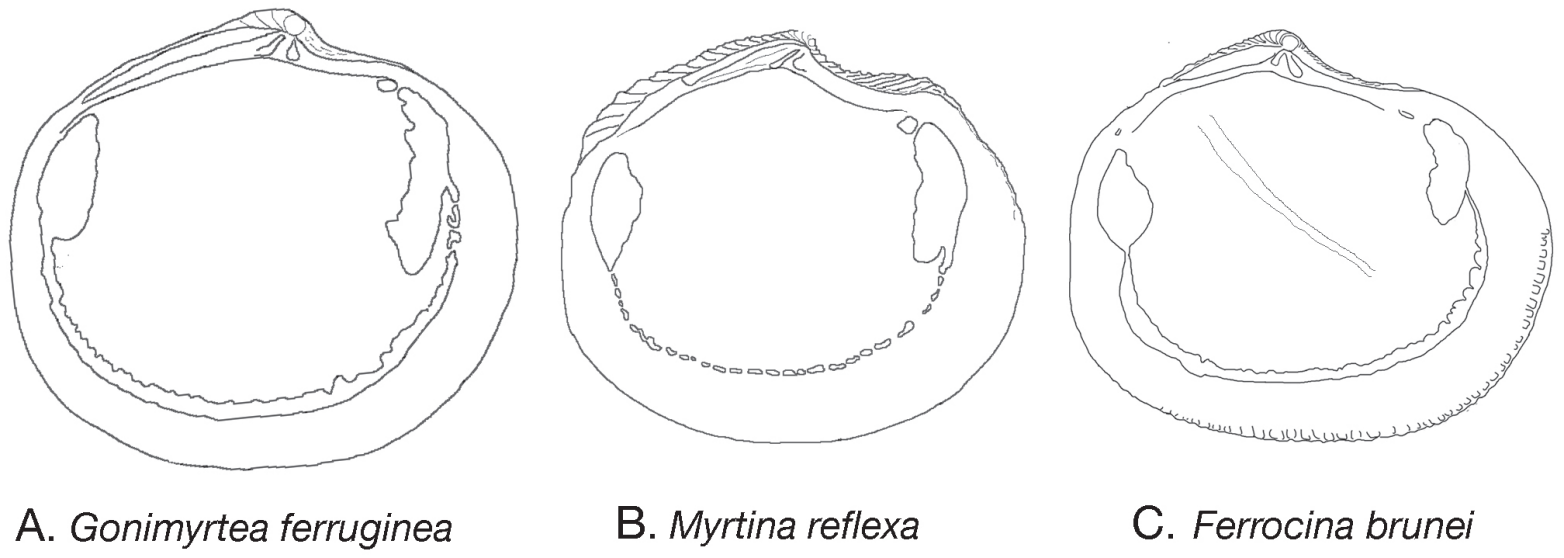
Internal outline drawings of **A** *Gonimyrtea ferruginea* **B** *Myrtina reflexa* **C** *Ferrocina brunei*. Not to scale.

##### Distribution.

Known only from type locality (Fig. 4).

##### Etymology.

Named for Sultanate of Brunei. Noun in apposition.

##### Remarks.

*Ferrocina brunei* is similar to the type species *Ferrocina mutiradiata* from Fiji but has much less prominent radial ribs, a less strongly denticulate shell margin and is smaller (shell length to 9 mm compared to 18 mm).

The anatomy is similar to most Lucinidae and bacterial symbiosis is confirmed by the presence of abundant bacteria in the ctenidial filaments. A distinctive feature of the anatomy is the bilateral ramshorn-like extension of the visceral mass anterior to the foot. We have observed similar structures only in *Bathyaustriella thionipta* Glover, Taylor & Rowden, 2004 from a hydrothermal vent on the Kermadec Ridge and in a *Ferrocina* species recently discovered off the southern USA in the Western Atlantic (unpublished observations). The function of this structure is unknown but thin sections of the structure in *Bathyaustriella thionipta* showed that it consisted of diverticula of the digestive gland.

### Subfamily Leucosphaerinae Taylor et al., 2011

#### Genus Gonimyrtea

Marwick, 1929

http://species-id.net/wiki/Gonimyrtea

##### Type species.

*Loripes concinna* Hutton, 1885. Original designation.

##### Definition.

Shells small, subcircular to ovate, higher than long, inflated. Sculpture of closely spaced thin, low commarginal lamellae. Right valve with single cardinal tooth and small anterior and posterior laterals sometime present; left valve with two cardinal teeth and small anterior and posterior laterals sometimes present. Lunule narrow lanceolate. Ligament short, curved. Anterior adductor scar detached for about 1/5–1/2 of length.

##### Remarks.

There has been much confusion concerning the concept of this genus following from Chavan’s (1969) illustration of an Eocene species with little resemblance to the type and also his assignment of *Alucinoma* as a synonym (see Glover and Taylor 2007). We originally placed this genus (based on shell morphology) in the Myrteinae (Taylor et al. 2011) but continuing molecular analysis (species described below as UGS-3 in Taylor et al. 2011 and unpublished data) suggests that it should be classified in the sub-family Leucosphaerinae, although the type species has not yet been investigated. As well as the genotype *Gonimyrtea concinna* from New Zealand we include *Gonimyrtea avia* and *Gonimyrtea fidelis* from around New Caledonia (Glover and Taylor 2007) and two new species from the Philippines (Glover and Taylor in press).

#### Gonimyrtea ferruginea sp. n.

http://zoobank.org/4BD0BF6A-71CE-47B0-8C70-5D0D2D148BFB

http://species-id.net/wiki/Gonimyrtea_ferruginea

Figs 9A , 10

Gonimyrtea ferruginea UGS-3, unidentified genus & species - Taylor et al. 2011, fig. 7J.

##### Type material.

*Holotype*: paired valves, live collected (MNHN IM-2009-10376) L 24.5 mm H 23.2 mm T whole shell 15.0 mm. *Paratypes*: 1 paired valve (MNHN IM-2009-10376) L 24.0 mm H 22.0 mm T 6.5 mm (single valve); 1 pv (MNHN IM-2009-10376) L 15.9mm H 14.2 mm T 7.7 mm.

##### Type locality.

New Caledonia, Chesterfield Bank, 19°35'S, 158°48'E, 680–722 m. N/O Alis campagne EBISCO 2005 stn CP2614.

##### Material examined.

All MNHN material from several deep water cruises in South west Pacific (for details see - www.mnhn.fr/musorstom/). **New Caledonia**, MUSORSTOM 4: 1 valve,stn CP158, 18°49'S, 163°15'E, 625 m. 2 valves, stn CP179 18°57'S, 163°14'E, 475 m.1 paired valve, stn CP 236, 22°11'S, 167°15'E, 495–550 m. BATHUS 2: 1 paired valve, stn DW740, 22°36'S, 166°27'E, 570-605 m. BATHUS 4: 1 valve, stn CP 900, 20°17'S, 163°50'E, 580 m. 2 valves, stn DW 908, 18°58'S, 163°11'E, 502–527m. 1 valve, stn CP905, 19°02'S, 163°16'E, 294–296 m. 1 valve, stn CP954, 21°44'S, 166°36'E 250–255 m. BIOCAL: 1 valve, stn DW40, 22°55'S, 167°24"E, 650 m. **Chesterfield Bank**, MUSORSTOM 5: 5 valves, stn 380, 19°38'S, 158°44'E, 555–570 m. 24 valves, stn 381, 19°38'S, 158°47'E, 620 m. **Coral Sea, Kelso Bank,** MUSORSTOM 5: 2 valves, stn 284, 24°10'S, 159°33'E, 225-230 m. **Vanuatu,** MUSORSTOM 8: 1 valve, stn DW1072, 15°40'S, 167°20'E, 622–625 m. **SW Pacific, Wallis Island,** MUSORSTOM 7: 3 valves, stn DW526, 13°13'S, 176°15'E, 355–360 m. 5 valves, stn DW527, 13°24'S, 176°15'W 540–560 m. 10 valves, stn DW 528, 13°24'S, 176°13'W, 435–515 m. 8 valves, stn DW601, 13°19'S, 176°17'W, 350 m. **Bank Bayonnaise,** MUSORSTOM 7: 1 valve, stn DW626, 11°54'S, 179°32W 597–600 m. 2 valves, stn CP629, 11°54'S, 179°32'W, 400–420 m. **Tuscarora Bank,** MUSORSTOM 7: 35 valves, stn DW556, 11°49'S, 178°18'W, 440 m.

##### Description.

**Figure 10.**
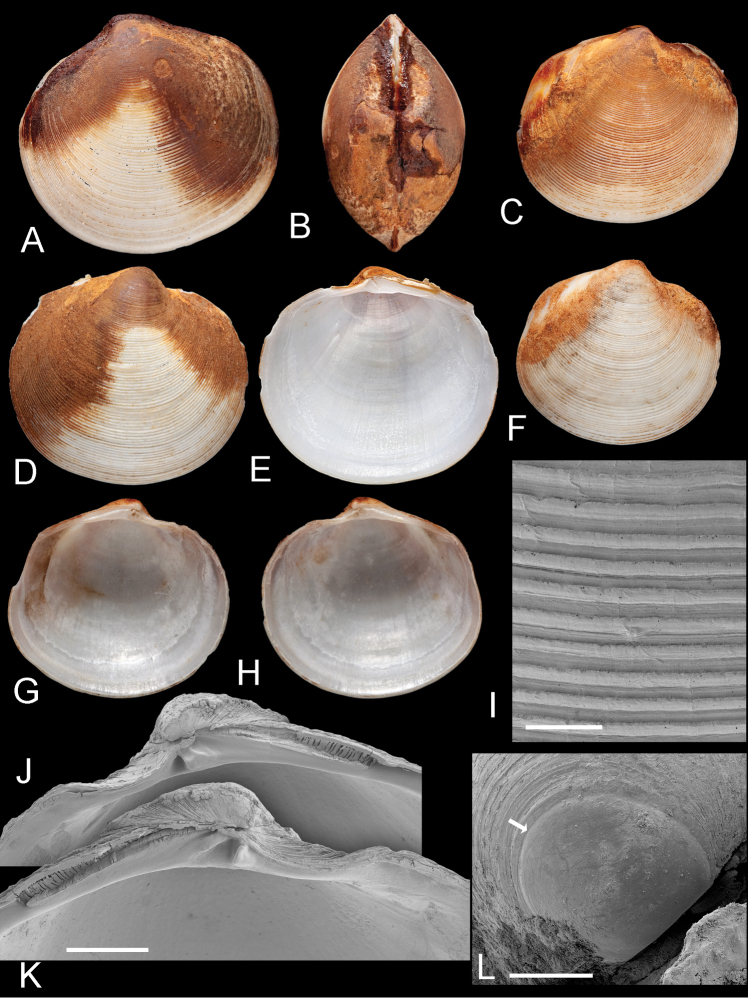
*Gonimyrtea ferruginea* new species. **A** Holotype, MNHN IM-2009-10376 exterior of left valve. L = 24.5 mm **B** Holotype, dorsal view **C** Paratype MNHN IM-2009-10376 exterior right valve L = 15.9 mm **D–E** Paratype MNHN IM-2009-10376 exterior and interior of right valve, sequenced specimen L = 24.0 mm **F–H** Paratype MNHN XXXXXXX exterior of right valve and interior of left and right valves. New Caledonia MUSORSTOM 4 stn CP236, 495–550 m, L = 12.5 mm **I–L** from BATHUS 2: stn DW740, 22°36'S, 166°27'E, 570–605 m**I** Detail of sculpture, scale bar = 500 µm **J, K** Details of hinge teeth right and left valves. Scale bar = 1 mm. **L** Protoconch. Arrow marks boundary between PI and PII. Scale bar = 100 µm.

Shell white, L to 25 mm H to 23.5 mm;subcircular, H/L 0.91 ± 0.017 (n=10), inflated T/L 0.28 ± 0.02 (n=10), sculpture of fine, low, rounded, regular, closely spaced, commarginal lamellae, radial sculpture absent, dorsal areas characteristically with ferruginous encrustations. Lunule short, lanceolate, symmetric. Protoconch (Fig. 10L): PI+PII 275 µm, PII, a narrow, 19 µm rim with fine increments. Ligament inset in narrow groove. Hinge (Figs 10J, K) LV with 2 cardinal teeth the posterior larger, can be obscure, lateral teeth not visible, RV with 2 cardinals, very small anterior lateral tooth, posterior lateral absent. Anterior adductor muscle scar long detached for ½ length, at an angle of 10–15°. Posterior adductor scar ovoid. Pallial line continuous, shell margin smooth.

##### Distribution.

New Caledonia, Vanuatu, and banks and islands in SW Pacific, in deeper water between 350 and 650 m (Fig. 4).

##### Remarks.

Compared to the genotype, *Gonimyrtea concinna* from New Zealand, at depths to 200 m, *Gonimyrtea ferruginea* occurs in much deeper water, is larger, the lateral teeth are less distinct, and commarginal lamellae are more closely spaced. Another deeper water species of *Gonimyrtea* is known from the Philippines (Glover and Taylor in press) occurs at depths of 500–1000 m but is smaller than G. *ferruginea*, more elongate, lamellae are narrower, the interspaces wider, and the lunule deeper and longer.

Both *Gonimyrtea ferruginea* and the new species from the Philippines typically have dense iron-rich encrustations on the dorsal areas of the shell (Fig. 10A–D). This also occurs in *Dulcina guidoi* from central Philippines at similar depths (Cosel and Bouchet 2008) but is an unusual feature of lucinids that otherwise often exhibit some brown staining around the anterior inhalant area and posterior apertures.

Molecular analyses show that *Gonimyrtea ferruginea* (as UGS-3 in Taylor et al. 2011) aligns in the subfamily Leucosphaerinae close to species of *Dulcina*. GenBank numbers FR 686701, FR 686779, FR 686606

##### Etymology.

Derived from Latin *ferrugineus* meaning rust-coloured. Adjective in nominative singular.

#### Genus Myrtina

Glover & Taylor, 2007

http://species-id.net/wiki/Myrtina

##### Type species.

*Myrtina porcata* Glover & Taylor, 2007 original designation.

##### Diagnosis.

Shell small, L to 16 mm, subcircular, posterior margin quadrate, sculpture of commarginal lamellae, elevated commarginal lamellae on dorsal margin, radial sculpture absent, lunule often strongly asymmetric, hinge with cardinal teeth in both valves, lateral teeth strong to obscure with RV anterior lateral tooth generally larger, anterior adductor muscle scar short, detached for about 1/5 length, pallial line either entire or in close, small blocks.

##### Remarks.

Eight species of *Myrtina* are known from the central IWP from shallow offshore to deeper water (Glover and Taylor 2007, in press; Fengshan 2011), including the Japanese species formerly known as *Lucinoma adamsiana* Habe, 1958.

#### Myrtina reflexa sp. n.

http://zoobank.org/C5254102-0F66-41A0-9A3E-8DB483BB7D44

http://species-id.net/wiki/Myrtina_reflexa

Figs 9B , 11

Lucina inanis (Prashad 1932) - Knudsen 1967: 286, pl. 2 figs 9,11. (non Prashad 1932).
Lucina inanis Prashad - Cosel and Bouchet 2008: 188, figs 55 A–F.

##### Type material.

*Holotype*: 1 whole shell, L 10.5 mm, H 9.4 mm, T 2.5 mm, NHMUK 1968738, John Murray Expedition stn 125, 825m, 5°36'12"S, 39°28'24"E off Zanzibar.

**Figure 11.**
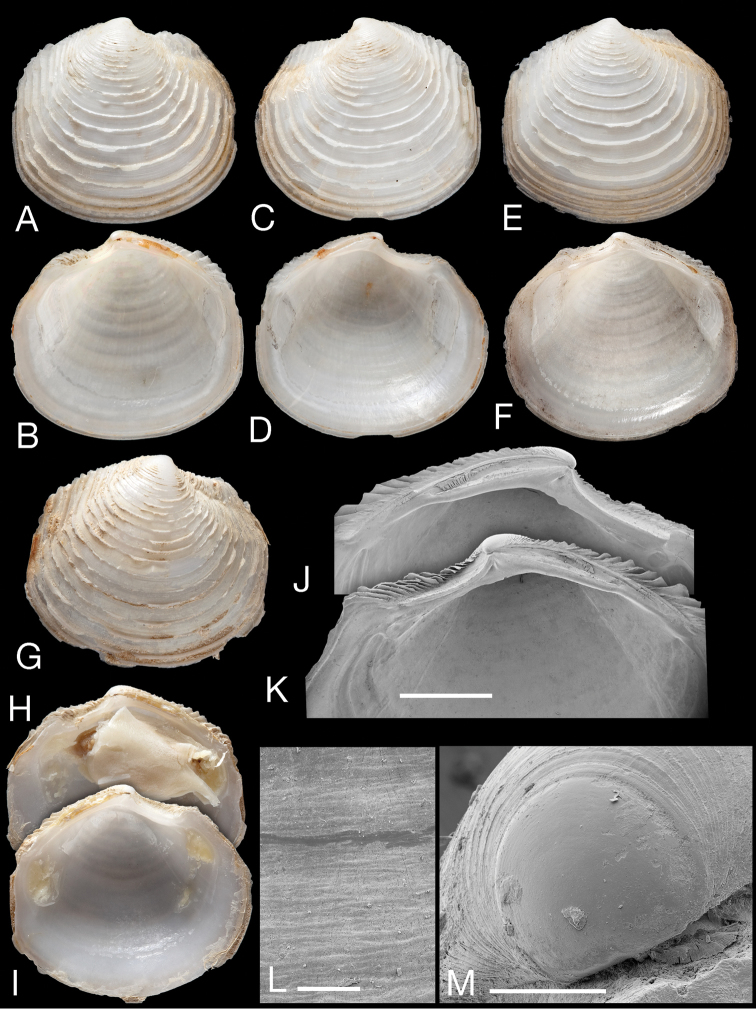
*Myrtina reflexa* new species. **A–D** Holotype, NHMUK 196838 exterior and interior of right (**A–B**) and left valves (**C–D**), L = 10.5 mm **E–F** Paratype NHMUK 20130121 John Murray Expedition stn 106, exterior and interior of right valve. L = 15 mm **G–I** Exterior of right valve and interior of right and left valves, MNHN IM 2009-8733, MIRIKY stn DW3239, 14°30'S, 47°26'E, 230–288 m, off NW Madagascar, L = 14.2 mm**J–K** Details of hinge of holotype left and right valves. Scale bar = 2.0 mm **L** Detail of microsculpture, holotype. Scale bar = 50 µm **M** Protoconch of holotype. Scale bar = 100 µm.

*Paratypes*: 2 RV, L 10.7 mm H 9.5 mm T 2.5 mm, L 11.8 mm H 10.7 mm T 2.8 mm, NHMUK 1968738 locality as holotype; 17 valves (13 RV, 4 LV), figured paratype (Fig. 11E-F) RV, L 15 mm, H 13.3 mm, NHMUK 20130121, John Murray Expedition stn 106, 183–194 m, 5°38'54"S, 39°18'42"E, off Zanzibar.

##### Other material.

1 whole shell (live collected) L 14.2 mm, H 12.7 mm, T 3.3 mm, MNHN IM 2009-8733, MIRIKY stn DW3239 14°30'S, 47°26'E, 230–288 m, off NW Madagascar.

##### Description.

Shells greyish white, H to 13.5 mm, L to 15.2 mm, T 3.8 mm, longer than high H/L = 0.91 ± 0.038 (n=8), T/L = 0.24 ± 0.018 (n=8), subovate, posterior truncate, anterior dorsal area marked by shallow double sulcus, anterior dorsal margin with elevated lamellae, umbones low, sculpture of widely spaced commarginal lamellae that are reflected ventrally, no radial sculpture. Microsculpture of fine growth increments (Fig. 11L). Lunule lanceolate, strongly asymmetric with larger part in LV. Protoconch (Fig. 11M): PI+ PII 183 µm, PII a narrow rim. Hinge (Figs 11J, K): LV with 1 thin cardinal tooth, laterals absent, RV with small, single, cardinal tooth, small anterior lateral tooth, posterior lateral teeth absent. Anterior adductor scar short, detached from pallial line for ¼ of length, posterior scar ovoid. Pallial line discontinuous in short narrow blocks. Shell interior glossy, shell margin smooth.

##### Distribution.

Western Indian Ocean, off Zanzibar and Madagascar from 200–825 m (Fig. 4).

##### Etymology.

Latin *reflexa* means bent or turned back, a reference to the form of the commarginal lamellae. Adjective nominative singular

##### Remarks.

This species was referred to *Lucina inanis* (Prashad) by Knudsen (1967) and the same specimens were figured and briefly discussed by Bouchet and Cosel (2008) who thought they more closely resembled species of *Alucinoma*. The type and other material of *Lucina inanis* from Indonesia and Philippines differ in shape and lack the regular, reflexed commarginal lamellae (Glover and Taylor in press). Characters of the new species suggest a placement in *Myrtina* and this position is corroborated by molecular evidence that places *Myrtina reflexa* in the subfamily Leucosphaerinae close to species UGS -1(now recognised as a new *Myrtina* species) from the Philippines (Taylor et al. 2011). *Myrtina* occur in offshore habitats from 30–1200 m from the Philippines and New Caledonia and are likely present in organically enriched sediments throughout the tropical Indo-West Pacific.

*Myrtina reflexa* differs from the genotype, *Myrtina porcata*, by the more widely spaced and reflexed commarginal lamellae. It is most similar to a new *Myrtina* species (Glover and Taylor in press) from 200–1200 m around the Philippines but is larger and has more prominent and reflexed commarginal lamellae.

## Supplementary Material

XML Treatment for Scabrilucina

XML Treatment for Scabrilucina victorialis

XML Treatment for Scabrilucina vitrea

XML Treatment for Scabrilucina melvilli

XML Treatment for Ferrocina

XML Treatment for Ferrocina brunei

XML Treatment for Gonimyrtea

XML Treatment for Gonimyrtea ferruginea

XML Treatment for Myrtina

XML Treatment for Myrtina reflexa
